# Neurofibroma Development in Neurofibromatosis Type 1: Insights from Cellular Origin and Schwann Cell Lineage Development

**DOI:** 10.3390/cancers14184513

**Published:** 2022-09-17

**Authors:** Ling-Ling Ge, Ming-Yan Xing, Hai-Bing Zhang, Zhi-Chao Wang

**Affiliations:** 1 Department of Plastic and Reconstructive Surgery, Shanghai Ninth People′s Hospital, Shanghai Jiao Tong University School of Medicine, Shanghai 200011, China; 2 CAS Key Laboratory of Nutrition, Metabolism and Food Safety, Shanghai Institute of Nutrition and Health, University of Chinese Academy of Sciences, Chinese Academy of Sciences, Shanghai 200011, China

**Keywords:** neurofibromatosis type 1, cutaneous neurofibroma, plexiform neurofibroma, development, cellular origin, Schwann cell lineage

## Abstract

**Simple Summary:**

Neurofibromas have been thought to originate from cells within the Schwann cell lineage, while no consensus has been reached so far about the specific time of initiation and the exact cellular origin. Moreover, the role of Schwann cell lineage transition in different developmental stages of neurofibromas, together with other determinant factors, remains controversial, despite intensive studies. In this review, we summarized the accumulating evidence about the full range of neurofibroma development based on cellular and molecular pathogenesis.

**Abstract:**

Background: Neurofibromatosis type 1 (NF1), a genetic tumor predisposition syndrome that affects about 1 in 3000 newborns, is caused by mutations in the *NF1* gene and subsequent inactivation of its encoded neurofibromin. Neurofibromin is a tumor suppressor protein involved in the downregulation of Ras signaling. Despite a diverse clinical spectrum, one of several hallmarks of NF1 is a peripheral nerve sheath tumor (PNST), which comprises mixed nervous and fibrous components. The distinct spatiotemporal characteristics of plexiform and cutaneous neurofibromas have prompted hypotheses about the origin and developmental features of these tumors, involving various cellular transition processes. Methods: We retrieved published literature from PubMed, EMBASE, and Web of Science up to 21 June 2022 and searched references cited in the selected studies to identify other relevant papers. Original articles reporting the pathogenesis of PNSTs during development were included in this review. We highlighted the Schwann cell (SC) lineage shift to better present the evolution of its corresponding cellular origin hypothesis and its important effects on the progression and malignant transformation of neurofibromas. Conclusions: In this review, we summarized the vast array of evidence obtained on the full range of neurofibroma development based on cellular and molecular pathogenesis. By integrating findings relating to tumor formation, growth, and malignancy, we hope to reveal the role of SC lineage shift as well as the combined impact of additional determinants in the natural history of PNSTs.

## 1. Introduction

Neurofibromatosis type 1 (NF1), also known as von Recklinghausen’s disease, is one of the most prevalent genetic tumor predisposition disorders. Individuals with NF1 are born with autosomal dominant mutations of a large tumor suppressor gene *NF1*, which encodes the neurofibromin protein. Neurofibromin is a GTPase-activating protein that downregulates the Ras signaling pathway [1]. NF1 affects about 1 in 3000 live births worldwide, without gender or racial preference, and causes a variety of clinical features involving various organ systems [2]. These manifestations include pigmented lesions (café-au-lait macules and axillary freckling), optic damage, visceral dysfunction, and skeletal dysplasia, as well as cognitive and behavioral impairments. The multisystem functional involvement of the *NF1* gene and the corresponding distinctive manifestations in NF1 individuals point toward strong genotype–phenotype correlations [3].

One of the hallmarks of NF1 is the development of peripheral nerve sheath tumors (PNSTs). These are known as neurofibromas and are composed of a mixture of nervous and fibrous tissue, such as Schwann cells (SCs), fibroblasts, endothelial cells, mast cells, macrophages, neurons, and extracellular matrix (ECM). According to their distinctive locations and timing of emergence, neurofibromas can be classified into two main subtypes: cutaneous/dermal and plexiform lesions [4].

Cutaneous neurofibromas (cNFs) are confined to nerve terminals in the dermis and occur in almost all patients with NF1, causing itching or stinging sensations. They typically emerge around puberty and increase in number, potentially reaching thousands of tumors over a lifetime (especially in pregnant women, as a result of regulation by sex hormones) [5,6]. Although similar to cNF at the histological level, plexiform neurofibromas (pNFs) congenitally grow along nerve plexuses with a rich vascular supply and involve multiple nerve fascicles, appearing in nearly 30% of NF1 patients [4]. In a longitudinal study analyzing the natural history of pNF, variable growth dynamics were seen in different age groups, among which the fastest growth rate (≥20% per year) occurred in patients under 5 years of age [7]. Growing pNFs can put pressure on the surrounding tissues, resulting in severe pain, neurological damage, and skeletal destruction. In addition, there is an approximately 10% lifetime risk of pNFs transforming into NF1-related malignant PNSTs (MPNSTs) [8,9,10]; these often arise within pre-existing pNFs, rather than cNFs, as a result of additional genetic mutations occurring in a subset of key genes in a specific order [11]. Given the occurrence of tumors at two distinct developmental stages (adolescent versus embryonic), in different locations (body surface versus nerve plexus), with differing malignant transformation potential (none versus 10%), the spatiotemporal heterogeneity of cNF versus pNF reasonably supports distinct cellular origins for these neurofibromas.

While, for decades, neurofibromas have been believed to originate from cells within the SC lineage [12,13], the specific time of initiation and exact cellular origin of pNFs and cNFs remain controversial, despite intensive studies. With the evidence that clinical presentation can differ substantially depending on the spatiotemporal somatic mutation of the *NF1* gene in certain cell types within the SC lineage, recent studies using genetically engineered mouse (GEM) models have shed light on the development of SC lineages. This has aided the clarification of neurofibroma characteristics with regard to formation, progression, and transformation to malignancy [14]. In this review, we summarize the accumulating advances in the understanding of the specific features of the different developmental stages of PNSTs, based on cellular and molecular pathogenesis perspectives.

## 2. Neurofibroma Formation

### 2.1. The Developmental Origin of SC Lineages

Friederich von Recklinghausen initially coined the concept of neurofibroma in 1882 [15], noting that both neuronal and fibrotic components were present within these tumors. In subsequent studies, the identification of abnormal SC proliferation in neurofibromas led to the SC origin hypothesis [16]; therefore, neurofibromas have long been recognized to originate from SC lineages. Despite the early consideration of mature SCs as the pathogenic origin, studies published recently following the establishment of various GEM models indicate the possibility that neurofibromas may originate from earlier-stage SCs [17,18,19,20,21]. To date, the specific cell type within the SC lineage leading to neurofibroma formation is controversial.

The term neural crest stem cell (NCSC) was first put forward by Stemple and Anderson in 1992, following their successful isolation of neural crest cell populations with self-renewal ability and multipotency in vitro [22]. NCSCs are a transient cell population, emerging at the dorsolateral portion of the neural tube during vertebrate embryogenesis and then migrating to extensive locations. They later differentiate into a wide range of cell lineages and tissues, depending on the local environment, including most of the neuronal and glial components of the peripheral nervous system (PNS), as well as bone, cartilage, endocrine cells, melanocytes, fibroblasts, and smooth muscle cells [23,24,25].

In the first stage of SC lineage development, a subpopulation of NCSCs gives rise to boundary cap (BC) cells. These are transiently located at the motor exit point (MEP) and the dorsal root entry zone (DREZ), acting as a boundary between the central and peripheral nervous systems and allowing the passage of axons [26,27]. The discovery of specific molecular markers has greatly contributed to the further characterization of BC cells [28]. These cells express the transcription factor gene *Krox20*, also known as *EGR2* in humans, and produce the SC components of the dorsal and ventral nerve roots, playing a role in the early myelination of the PNS [29]. Moreover, in culture, BC cells can also generate other cell types, such as melanocytes, astrocytes, and neurons [28,30]. In addition, a subpopulation of BC derivatives was recently found to express *Prss56*; lineage-tracing studies demonstrated that *Prss56*-expressing BCs have broad differentiation potential and can give rise to SCs in the nerve roots, hypodermis, and dermis, suggesting the potential of BC cells as candidates for the cellular origin of both pNFs and cNFs [31]. The specific expression pattern of *Krox20/EGR2* and *Prss56*, together with *Hey2* and *Wif1* in mouse and/or human lines, suggests that BC clusters emerge at embryonic day (E) 10.5–11 in mice [32].

In addition to differentiation into BC cells, migrating NCSCs (both multipotent and restricted) can differentiate into Schwann cell precursors (SCPs) at around E12 to E13 in mice [33]. Furthermore, both *Krox20*-expressing and *Prss56*-expressing BC cells can convert to SCPs in nerve roots and to satellite cells and nociceptive neurons in the dorsal root ganglia (DRG) [28,34,35]. SCPs are glial-restricted cells found in early embryonic nerves, which are in intimate contact with nerve axons and maintain a certain level of multipotency; they have the ability to generate endoneurial fibroblasts, melanocytes, and parasympathetic or enteric neurons. Although they share some common features with NCSCs, SCPs differ in the expression of specific glial differentiation genes and molecular markers, such as myelin protein 0 (P0), growth-associated protein 43 (GAP43), cadherin-19, and other molecular factors [36]. Another specific characteristic of SCPs is their dependence on axon-associated signals, which determine their proliferation and differentiation to myelinating or non-myelinating cells [37]. In the second stage of SC lineage development, a subset of SCPs converts into immature SCs at E13–15 in mice, regulated by a number of signals associated with axons, including neuregulin 1 (NRG1), endothelin, and the notch signaling pathway. Similar to SCPs, immature SCs maintain close contact with axons but differ substantially in their molecular phenotype, with increased expression of specific proteins, including glial fibrillary acidic protein (GFAP) and S100 calcium-binding protein (S100). In addition, the survival of immature SCs depends on autocrine signals, rather than axon-associated NRG1 signals.

**Figure 1:**
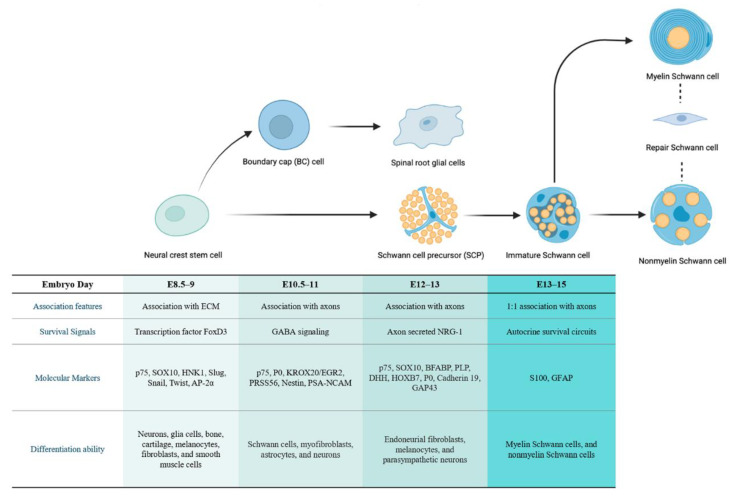
The developmental stage of SC lineage and corresponding characteristics of different cell types. Neural crest stem cells (NCSCs) can differentiate into multipotent boundary cap (BC) cells and SC precursors (SCPs). The SCPs further develop into immature SCs, which then differentiate into myelinating/non-myelinating SCs according to the associated axons. These mature types can de-differentiate upon specific mutation or injury into repair SCs. The corresponding embryogenesis time of each cell type in mice and other features, including their association characteristics, survival signals, molecular markers, and differentiation capacity, are listed relative to the cells.

In the subsequent stage, the associated axons determine the developmental type of immature SCs [38]. Immature SCs that are in contact with large-diameter axons, reaching a ratio of 1:1 through proliferation, and proceed to transform into myelinating SCs (mSCs) around birth [39]. In contrast, immature SCs in contact with small-diameter axons develop into mature non-myelinating SCs (nmSCs) at varying SC-to-axon ratios and form Remak bundles [37] (Figure 1).

#### 2.1.1. The Cellular Origin of Neurofibroma

The cutaneous form of NF occurs in almost all NF1 patients, with tumors typically emerging around puberty and potentially increasing in number over the lifespan of the patients. In contrast, pNFs arise in around 30% of NF1 patients from early childhood and gradually expand throughout life. The significant differences between these two subtypes of neurofibromas and the phenomenon that mouse models develop pNF but fail to develop cNF at 100% frequency jointly indicate that the cellular origins of these lesions may differ. Specifically, their temporally and spatially distinct clinical characteristics support the hypothesis that pNFs are congenital lesions arising from the embryonic SC lineage, whereas cNFs likely derive from a more mature cell type in the SC lineage [40]. The study progress of the cellular origin of both pNF and cNF is summarized in Table 1.

#### 2.1.2. The Cellular Origin of pNF

Although the hypothesis of the SC origin of neurofibroma has been put forward by researchers for decades, it was not until 2002 that GEM models successfully recapitulated human pNF lesions, definitively demonstrating the potential of SCs to be the lineage of origin. Knowing the crucial role of *Krox20* in SC development, Zhu and coworkers used *Krox20*-*Cre* in mouse models to specifically delete *Nf1* in SC lineage cells [12]. They found that loss of *Nf1* from the SC lineage in an *Nf1^+/−^* environment successfully recapitulated pNF formation in spinal nerve roots. However, although *Krox20*-*Cre* could induce pNFs, the extensive expression of *Krox20* in NCSCs, SCPs, and SCs meant that the exact time of initiation and cells of origin remained unknown [29]. In 2008, Joseph et al. showed that germline deletion or conditional deletion of *Nf1* using *Wnt1*-*Cre* led to transient hyperproliferation and self-renewal of NCSCs without typical tumor formation. In addition, no NCSCs were identified in normal adult peripheral nerves or the regions that develop neurofibroma, and no tumorigenicity due to *Nf1* loss in NCSCs was observed. Accordingly, the authors speculated that neurofibromas might arise from later NCSC derivatives [41]. In the same year, Zheng et al. induced mutation of *Nf1* in SCPs using *P0a-Cre* rather than the *Krox20*-*Cre*, which led to pNF formation in the sciatic nerve. The results suggested that nmSCs of the Remak bundles might be the cellular origin for neurofibroma [42]. However, no conclusion could be drawn as to which stage in the SC lineage was critical for neurofibroma formation mediated by *NF1* loss. In 2011, Le and colleagues reported that inducible *Plp*-*CreER^T2^*-mediated ablation of *Nf1* in SCs during both embryonic and adult stages resulted in peripheral nerve hyperplasia and pNF formation. However, embryonic stages (including SCPs and immature SCs) were more susceptible to pNF, in comparison with adult stages (100% versus 2%) [17]. Another study, carried out by Mayes and coworkers, proposed that embryonic and adult SCs had similar potential to give rise to neurofibromas; however, the clinical manifestation of pNFs as congenital lesions is less supportive of a central role for mature SCs [18]. In 2014, Chen et al. reported that the cells of origin for paraspinal pNF were PLP^+^GAP43^+^ cells, which could be detected in the embryonic DRG at E11.5 but not at E13.5. It was also demonstrated that PLP^+^ cell populations included both embryonic Krox20^+^ and Dhh^+^ cells [20]. Due to their specific expression of molecular markers, PLP^+^GAP43^+^ cells were considered to be at the SCP developmental stage and therefore potentially the elusive cells of origin for paraspinal pNF. The authors hypothesized that there may be an overlapping of cell types in the transition from NCSCs to embryonic and mature SCs, such that a subpopulation of the remaining SCPs could continue into adulthood and retain the potential for pNF formation [20].

#### 2.1.3. The Cellular Origin of cNF

Unlike the considerable achievements made in developing GEM models to study the cellular origin of pNF, few animal models have been established to recapitulate the characteristics of cNF, leaving its origin and pathogenic mechanisms relatively unknown. Given the near 100% incidence of cNF in NF1 individuals, there remains an urgent need to investigate the formation and development of cNF. The first GEM model to successfully generate cNF was produced by Satio et al. in 2007, using *Camk2*-*Cre* to drive N-Ras activation [44]. These transgenic mice exhibited hyperpigmentation of the epidermis throughout their lives and developed diffuse cNF later on. Nonetheless, pNF lesions and other manifestations, such as schwannomas and astrocytomas, were not detected in this study. The authors speculated that further signals in addition to activated N-Ras may be required for the development of these tumors. In 2008, Wu and colleagues established a GEM model using *Dhh*-*Cre* to inactivate the *Nf1* gene [45]. In vivo ablation of *Nf1* at E12.5 not only recapitulated human pNF but also effectively generated cNF in an *Nf1^+/−^* microenvironment. The results obtained in these studies overturned the previous view that cNF probably arose from mature cell types in the SC lineage, based on its time of initiation [40]. Regarding the location of cNF, follow-up studies further explored its specific origin, focusing on another stem cell population known as skin-derived precursors (SKPs), found in the dermis of humans and mice. SKPs are also multipotent, with the capacity to differentiate along neuronal and glial cell lineages, giving rise to SCs, neurons, adipocytes, and other cell types. In 2009, Le et al. pioneered research into the ability of SKPs to induce neurofibromas upon *Nf1* loss. In this study, SKPs isolated from tamoxifen-treated *Nf1^−/−^ CMV-CreER^T2^* mice that had been injected in the proximity of the sciatic nerves recapitulated pNF, indicating the intrinsic capacity of SKPs to generate neurofibromas. However, SKPs implanted in the dermis of mice could also generate classic cNF lesions [46]. These data suggested that a specific cell type within the SKP population was the cellular origin for cNF tumor initiation. However, since SKPs are a heterogeneous cell population, the essential questions of which subsets of cells give rise to which subtype of neurofibromas or whether there is a common origin within SKPs to form both cNFs and pNFs in the absence of *NF1* remain to be answered.

#### 2.1.4. Associate pNF and cNF with a Common Stage of Origin

With the discovery of SKPs as a possible common origin for the different subtypes of neurofibromas, the previous concept of distinctive initiation stages was transformed to that of a shared initiation stage. The explanation for the difference in the timing and location of occurrence was the spatiotemporal difference in *NF1* loss at subsequent developmental stages. In 2019, Chen and coworkers [14], as well as Radomska and colleagues, proposed that cNFs and pNFs may originate separately from the same cell population, *HoxB7*/*Prss56*-expressing BC cells or SCPs [47]. However, despite the effective generation of pNFs and cNFs in GEM models and breakthroughs in the hypothesis of cellular origin, the distinctive phenotypes observed in mouse models and human neurofibroma require further investigation. In 2021, Mo et al. used human induced pluripotent stem cells (hiPSCs) to identify the common cells susceptible to mutation in different types of neurofibromas [48]. The results suggested that biallelic inactivation of *Nf1* in SOX10^+^ cells of the SC lineage could lead to the formation of both cNFs and pNFs. Future investigations utilizing these hiPSC lines will allow the mechanisms that define neurofibroma formation to be better understood by applying the insights gained from studies into cellular origin.

### 2.2. Alterations in SCs in the Early Stage of Tumorigenesis

Under normal circumstances, SCs cover most of the surface of peripheral nerve axons, and their behavior is recognized to be adhesively controlled by axonal contact. Signals regulating survival, proliferation, and differentiation transmitted via axons during embryonic and adult stages are regarded as vital to maintaining SCs in a differentiated state and ensuring normal neural functions [38,49]. In recent years, the molecular mechanisms of SC–axonal interactions, including the NRG1-ErbB signaling pathway, have been widely studied.

Loss of contact between transiently proliferating SCs and axons is a common occurrence in the early stages of neurofibroma development [45]. A mechanistic explanation provided for this crucial event is that disruption to SC–axonal interactions results from the Ras-Raf-ERK-dependent downregulation of an SC surface protein named semaphorin 4F (Sema4F) [50]. High levels of Ras signaling and low levels of Sema4F trigger tumorigenic properties in neoplastic SCs, inducing increased proliferation. In addition to the molecular mechanisms of pNF, Radomska et al. provided a perspective on the occurrence of cNF [47]. They hypothesized that the increase in density of local innervation in mutant skin might be a mechanism to compensate for SC hyperplasia in order to maintain appropriate levels of contact; however, when overridden, SCs can no longer interact with axons, and the increased branching may lead to a pro-tumorigenic phenotype. The branching capacity of nerve terminals in the upper dermis may be associated with the lack of perineurium [47].

## 3. Neurofibroma Progression

### 3.1. SCs Contribution and Lineage Shift

In the process of neurofibroma growth and progression, SCs, the most abundant glial cells in the PNS and also the suspectable tumor cells of neurofibromas, have been shown to play multiple roles. Stonecypher et al. found that neoplastic SCs could produce NRG1, which then promoted neoplastic SC proliferation in an autocrine or paracrine way [51]. Neoplastic SCs also secreted cytokines, such as stem cell factor (SCF) and colony-stimulating factor 1 (CSF1); such factors were proposed to act in a “cytokine-cytokine receptor” manner, recruiting immune cells such as mast cells and macrophages, both of which secrete transforming growth factor-β (TGF-β) to active neurofibroma-associated fibroblasts for ECM remodeling [51]. As in the process of neurofibroma formation, a process of rapid de-differentiation of SCs is triggered by axonal damage, which subsequently destroys the myelin sheath. With the development and progression of the tumor, these SCs undergo consistent de-differentiation and finally revert to a progenitor-like state of proliferation [52]. In this process of cellular transition, the synergistic effects of the Ras-Raf-MEK-ERK pathway and inflammatory signals have been demonstrated as the driving factors [52]. Several studies have been explored to identify related inflammatory signals and determine altered gene expression patterns involved in this conversion process, including downregulation of genes coding for the key myelin transcription factor *Krox20*, as well as structural proteins such as *P0*, and upregulation of pro-inflammatory factors, such as tumor necrosis factor α (TNFα), interleukin-1α (IL-1α), and interleukin-1β (IL-1β) [47,53,54].

Specifically, additional effects of nerve injury in facilitating SC phenotype transition have also been recognized. To verify this, the researchers obtained pigmented melanocytes (probably by SC trans-differentiation) and rare neurofibroma formation after cutting the sciatic nerve in *Nf1* heterozygous mice [55]. Ribeiro et al. performed nerve crush in *P0*-*Nf1*^fl/fl^ and P0-*Nf1*^fl/^^−^ mice that do not develop neurofibromas, and observed infiltration of immune cells and appearance of neurofibromas [56].

**Figure 2:**
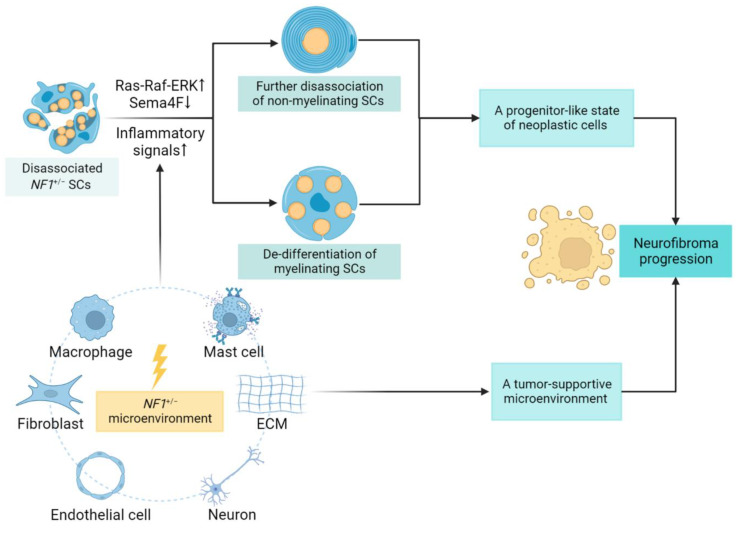
SC lineage shift and contributing factors in neurofibroma progression. The neoplastic SCs can rapidly de-differentiate to a progenitor-like state, disrupting SC–axonal interactions with tumor development. The underlying mechanism involves Ras-dependent downregulation of an SC surface protein, semaphorin 4F (Sema4F), together with elevated inflammatory signals, especially upon injury. Other environmental factors, including cellular and non-cellular components, further create a tumor-promoting microenvironment. The proliferative state of neoplastic cells and supportive tumor microenvironment combined to promote neurofibroma progression. ↑: upregulation of signaling pathways; ↓: downregulation in expression.

Wound repair following local trauma is regarded as a dynamic process followed by three main phases—inflammation, proliferation, and remodeling—in which various candidate mediators participate [57]. Thus, upon local trauma, the demand for new undifferentiated cells is met by the nerve regeneration capacity, which can promote the transformation of mSCs and Remak bundles into repair SCs, which is a pro-tumorigenic phenotype and capable of accelerating neurofibroma progression [52] (Figure 2).

### 3.2. Role of the Tumor Microenvironment

During the early embryonic stages, the microenvironment appears to be tumor-suppressive, allowing normal differentiation and proliferation of *NF^−/−^* SCPs [52]. However, as neurofibromas develop, the nerve microenvironment converts to a tumor-promoting type, with complex mutual interactions between cellular and non-cellular components. As heterogeneous tumors, neurofibromas comprise neoplastic SCs as well as fibroblasts, immune cells, neurons, endothelial cells, and ECM components. In addition to the original neoplastic cells, the non-neoplastic cell types in the tumor microenvironment are also crucial in the development of neurofibromas. A series of genetic studies have demonstrated that *NF1*-homozygous SC lineage cells and haploinsufficiency of *NF1* in non-neuronal cells are both required to promote the pathogenesis of neurofibroma [45,46,58,59,60,61]. The complex effects of the tumor microenvironment on neurofibroma formation and progression, especially the intricate interactions of both cellular and non-cellular components, have been summarized in detail in a review published in 2021 [62]; however, specific mechanisms remain unclear. Moreover, the occurrence of neurofibroma in normal individuals, as well as the recognition of patient subgroups with mosaic NF1 caused by postzygotic NF1 mutation, suggest that an *NF1^+/−^* environment may not necessarily be required for neurofibroma formation [45]. Thus, further studies and animal models are still urgently required to recapitulate the characteristics of the human neurofibroma microenvironment and shed light on its function in neurofibroma growth and progression.

## 4. Malignant Transformation of Neurofibroma

### 4.1. SCs Transition and Microenvironment Alteration

Transformation of neurofibromas to MPNSTs in NF1 patients occurs with a frequency of 8–16% [63], and benign and malignant lesions have distinct cellular and molecular characteristics, as well as different clinical and pathological behaviors [64]. With the neurofibroma–MPNST progression, the immunohistochemical characteristics suggest a dramatic change of molecular phenotypes referring to distinct SC compositions within the tumor. Lee et al. utilized microarray analyses to identify the abnormal profiles in an MPNST-derived cell line, T265, by comparing them with that of normal human SCs [65]. The findings indicated that widespread dysregulation of fundamental biological processes is essential for the proliferation and aggressiveness of malignant cells. As a molecular marker of SCs in neurofibroma, S100 protein (cytoplasmic and nuclear) and SOX10 (nuclear) expression are often reduced or even absent on progression to MPNST, which can be partially explained by a decrease in the proportion of differentiated SCs during malignant transformation [66]. In contrast to the low expression levels of CXCR4 and its ligands, CXCL12, in the embryonic SC lineage as the origin of *NF1*-deficient tumors, high levels in tumor cells from MPNST mouse models were measured by Western blotting. Moreover, the use of AMD3100 to antagonize CXCR4 was demonstrated to have proliferation-inhibitory effects on mouse and human MPNST cells [67]. Moreover, loss of CD34-positive fibroblastic network, together with changes in ECM components in comparison to benign lesions, could lead to altered cell–cell interactions within the tumor microenvironment, further promoting the development of MPNST [66,68].

### 4.2. Accumulation of Additional Gene Mutations

A second hit, also known as somatic mutation, inactivates the remaining wild-type copy of *NF1*, which is the main underlying mechanism for the initiation of PNSTs [69]. On this basis, malignant transformation is thought to be necessarily linked to additional gene mutations. Atypical neurofibromatous neoplasms of uncertain biologic potential (ANNUBP) are the precursor lesion of NF1-related MPNSTs, representing a pre-malignant state. Pemov et al. stated that deletion of the cell cycle regulator locus *CDKN2A/B*, along with loss of the *NF1* gene, is a required step for pNFs to develop into ANNUBPs and subsequently progress to MPNSTs [70]. In addition, copy number variation and mutations in the tumor suppressor gene *TP53* have been identified in some NF1-related MPNST cases [71]. The first GEM model of MPNST induced mutation of both *Nf1* and *Tp53* on chromosome 11 as the initiation event, which represented a milestone in the NF1 research field. However, subsequent studies showed a relatively low penetrance of *Tp53* gene changes (around 30%) in MPNST, indicating that it was not essential for all malignant cases [72,73]. Subsequently, a third hit is required to trigger MPNST formation, including the inactivation of polycomb repressive complex 2 (PRC2) subunits, i.e., the suppressor of zeste 12 homolog (*SUZ12*), and embryonic ectoderm development (*EED*) genes, occurring in approximately 70% of the NF1-related MPNSTs [74]. Apart from the typical ablation of tumor suppressor genes and loss-of-function of the core components for proper PRC2 function, other candidate genes have been proposed for NF1-related MPNST development, the most frequently reported ones including *SOX9/10*, *ERBB2/3*, *TWIST1*, *FGFR*, *EGFR1*, *PTEN*, *BRAF*, *TOP2A*, *KIT*, and *PDGFRA*. [75,76,77,78,79,80,81]. Despite extensive studies on the genetics of MPNSTs, a full understanding of their molecular diversity is lacking [11].

### 4.3. Dysregulated Signaling Pathways

Although tumor suppressor gene mutations play an important role in MPNST pathogenesis, it is likely that dysregulated signaling by as yet unidentified growth factors also contributes to the formation of these soft tissue sarcomas. In addition, overexpression of several growth factors and growth factor receptors that act as key upstream mediators of Ras activation has been suggested to play a vital role in promoting malignant transformation to MPNSTs [82]. NRG1 growth factors have been suggested as candidate promoters of mitogenesis in both neurofibromas and MPNSTs. Neoplastic SCs within these tumors variably co-express ErbB kinases (which mediate the NRG1 response) with upregulated NRG1, suggesting the promotion of autocrine or paracrine survival and proliferation signaling pathways in tumor cells [51,64,83]. EGFR, a membrane RTK closely related to the NRG1 receptors, has also been implicated in the pathogenesis of MPNSTs. Other factors or receptors, such as hepatocyte growth factor and its receptor c-MET [84,85], platelet-derived growth factor [86], TGF-β [87], insulin-like growth factor 1 receptor [88], midkine [89], lysophosphatidic acid [90], and stem cell factor and its receptor c-Kit [91], have also been reported to participate in the development of both neurofibromas and MPNSTs. In addition, critical signals, including MAPK, mTOR, and Wnt pathways, are regarded as core regulators of the progression from pNF to MPNST [92,93,94].

## 5. Discussion and Future Directions

In this review, we have collated evidence of the exceptional efforts and breakthroughs made in the study of the formation, progression, and malignant transformation of different types of neurofibromas in NF1 patients. Thanks to the successful establishment of various GEM models, as well as the recent application of hiPSCs to produce humanized models of NF1-associated neurofibromas, lesions completely recapitulating their human counterparts have been effectively generated for the investigation of intrinsic mechanisms. From the hypothesis of SC lineage to stem cells as early-stage tumor cells, the understanding of pNF and cNF initiation has evolved from the assumption of diverse stages to the notion of a common initiating stage, explained by subsequent spatiotemporal differences in *NF1* ablation. The wide range of tumor subtypes and their diverse locations support the concept of *NF1* loss in undifferentiated precursor cells during early developmental stages. However, there remains a view that the development of the SC lineage from NCSCs to immature stages and onward to maturity is not firmly governed by defined and periodic transitions. Instead, considerable overlap can be seen in developmental phases; the precursor stage of SCs can therefore persist into adulthood and retain the potential for neurofibroma formation. Nonetheless, no consensus has so far been reached about tumor initiation in neurofibroma cells. The facility for further investigation of specific cellular origin provides fertile ground for additional insights into the entire process of neurofibroma development from benign lesions to MPNSTs.

Apart from the essential role of neoplastic cells in tumor formation and progression, the cellular and non-cellular components of the tumor microenvironment are also regarded as indispensable elements in NF1-associated neurofibroma development. Various cell types, including inflammatory cells, fibroblast cells, endothelial cells, and others, are closely associated with the abnormally proliferating tumor cells through complex interaction mechanisms. Furthermore, ECM constituents, additional modifications, signaling pathways, and local trauma or injury, as well as specific hormones, can all exert considerable influence on the pathogenesis of neurofibroma [14,47,62]. Contrasting opinions about the necessity for an *NF1^+/−^* microenvironment in neurofibroma initiation and progression [45,46] show the urgent need for humanized and animal models to illustrate the role of the tumor microenvironment better. The classification of the mechanisms within each participating factor further requires the identification of molecular markers. Recently, Brosseau and colleagues were the first to perform single-cell RNA sequencing analysis using human neurofibroma samples to identify potential markers [95], providing new insight into the tumor microenvironment; however, there are still large gaps to fill in this field of research.

In addition, the contribution of nerves to the pathogenesis of various types of cancers has emerged as an important component in the tumor microenvironment and led to a new research hotspot [96,97,98]. As a defined PNST itself, the possible role of nerve tissue in the constitution of the microenvironment and the promotion of neurofibroma development, as well as specific mechanisms of action, remains poorly investigated. Liao et al. showed that *NF1*-deleted SKPs could give rise to neurofibromas only when injected into the periphery of the injured sciatic nerve [99]. Notably, they also established a three-dimensional skin raft culture using *NF1*^+/−^ nerve tissues together with *NF1*^+/−^ SKPs, thereby further supporting the hypothesis that nerves are essential environmental factors to facilitate neurofibroma development in subcutaneous tissues [99]. Recently, a mechanistic study conducted by Anastasaki et al. found elevated baseline neuronal excitability and deregulated hyperpolarization-activated cyclic nucleotide-gated (HCN) channel function in *NF1*-deleted sensory neurons. The subsequent collagen-type I alpha 2 chain (COL1A2) secretion leads to increased *NF1*-mutant SC proliferation and pNF growth [100]. However, this area still requires further research, focusing on the impact of nerve tissue as a regulator in the neurofibroma microenvironment and the role of the tumor microenvironment in recruiting nerves.

As malignant sarcomas of the soft tissue, NF1-associated MPNSTs tend to arise from the progression of patients with pNF rather than cNF; however, the underlying mechanisms are poorly understood. Although researchers have proposed the hypothesis that the unique susceptibility of pNFs to malignant transformation indicates that this subtype includes specific susceptible cell populations [11], no relevant models have been established, and there is little supporting evidence. In addition, the lack of comprehensive genetic data for numerous MPNST cell lines has largely hindered pathogenesis study and novel therapy development [101]. Tremendous efforts should be made to establish a detailed database, providing a platform for further research, such as genotype–phenotype correlation.

## 6. Conclusions

To conclude, the wealth of work exploring the pathogenesis of neurofibromas in NF1 individuals presented in this review has brought in-depth insights into the pathogenesis of the full range of benign tumors as well as MPNSTs. However, there is still a large gap in the existing understanding of many detailed aspects of neurofibroma development, as described above. Therefore, further clarification of cellular origin, the role of the tumor microenvironment, and mechanisms of malignant transformation will be of the utmost importance to enable the pathogenesis of neurofibroma to be expounded more fully. This will facilitate the discovery and evaluation of precise therapeutic targets in the near future.

## Figures and Tables

**Table 1:** The summarized study progress on the cellular origin of neurofibromas.

Study ID	Subtypes of NF	GEM Model	Cell of Origin	Supported Points	Unsupported/Unknown Points
Zhu et al., 2002 [12]	pNF	*Krox20-Cre*	SC lineage	Use of *Krox20-Cre* to ablate *Nf1* function within the SC lineage led to pNF.	The exact cellular origin remained unknown due to the extensive expression of *Krox20* in NCSCs, SCPs, and SCs.
Joseph et al., 2008 [41]	pNF	*P0a-Cre*	Later NCSC derivatives	Loss of *Nf1* function in NCSCs resulted in transient hyperproliferation instead of tumorigenesis. Neurofibromas may arise from differentiated cell types but not NCSCs.	The authors failed to detect the cellular origin of cNF, with no typical cutaneous lesions generated in any of the mouse models.
Zheng et al., 2008 [42]	pNF	*P0a-Cre*	nmSCs	The molecular signatures of the proliferating neoplastic cells were similar to nmSCs but not NCSCs.	The specific mechanism leading to the transformation of SCs from axon-associated to axon-disassociated cells in pNF remained unclear.
Le et al., 2011 [17]	pNF	*Plp-CreER^T2^*	SCPs and immature SCs	The embryonic stage showed enhanced susceptibility to pNF formation compared with the adult stage.	Another study showed that loss of *Nf1* at either embryonic or adult SC stages could lead to neurofibroma formation [18].
Maye et al., 2011 [18]	pNF	*Plp-Cre*	Embryonic/adult SCs	Loss of *Nf1* in either embryonic or adult SCs caused neurofibroma formation.	The capability of mature SCs to generate pNF was less supported by its clinical manifestation as a congenital lesion.
Keng et al., 2012 [19]	pNF	*Dhh-Cre*	SCs and SCPs	Loss of *Pten and Nf1 was* sufficient for progressing from pNFs to MPNSTs.	A previous study using the *mGFAP-cre* with conditional inactivation of both *Pten* and *Nf1* failed to develop neurofibromas [43].
Chen et al., 2014 [20]	pNF	*Plp-Cre*	GAP43^+^ PLP^+^ SCPs	GAP43^+^ PLP^+^ cells were detected in the embryonic nerve roots at E11.5, and acute loss of *Nf1* in SCPs led to pNF formation.	The remaining SCPs may persist into the adult stage and retain the capacity to form pNFs. However, the overlap of cell types in the transition from NCSCs to embryonic and mature SCs remained unknown.
Chaney et al., 2020 [21]	pNF	*Dhh-Cre*	Developing SCs	Loss of *Ink4a/Arf* in mice (*CDKN2A* in humans) and *Nf1* generated paraspinal neurofibromas and precursor malignant lesions.	Malignant transformation only occurred after transplantation into secondary mice, indicating the necessity of an immune microenvironment for tumor progression.
Saito et al., 2007 [44]	cNF	*Camk2-Cre*	Neural crest-derived cells	Activation of the N-Ras signaling pathway expressed in neural crest-derived cells caused cNF formation.	The differences between the Ras signals leading to cNF and pNF and the specific cell type of cNF origin remained unclear.
Wu et al., 2008 [45]	cNF, pNF	*Dhh-Cre*	SCP	Loss of *Nf1* in SCs at E12.5 was sufficient to give rise to both pNF and cNF in a wild-type microenvironment.	The cNFs observed in mouse models were found outside the dermis, below the panniculus carnosus, differing from the location in humans.
Le et al., 2009 [46]	cNF, pNF	*CMV-CreER^T2^*	SKP	The capability of SKPs to express *Dhh* and generate both pNF and cNF was identified.	Since SKPs are a heterogeneous cell population, the specific subpopulation acting as the cellular origin of cNF remained unknown. In addition, it was unclear whether there was a common cellular origin for cNF and pNF.
Chen et al., 2019 [14]	cNF, pNF	*Hoxb7-Cre*	*Hoxb7* lineage-derived cells	Loss of *Nf1* in *Hoxb7*-derived cells could recapitulate both pNF and cNF.	Loss of *N1* occurring before the bifurcation into distinct SC lineages and therefore giving rise to both cNF and pNF after subsequent differentiation was not definitively confirmed.
Radomska et al., 2019 [47]	cNF, pNF	*Prss56-Cre*	BC cells	BC-derived nmSCs and subepidermal SCs constitute the major population of pathogenic cells in pNF and cNF, respectively.	The differences in phenotypes between mouse models and human neurofibroma require further investigation.
Mo et al., 2021 [48]	cNF, pNF	SOX10^+^ cells	SOX10^+^ stem cells	Humanized models established using hiPSCs showed that inactivation of both *Nf1* alleles in mouse SOX10^+^ cells led to cNF and pNF formation.	This study further identified the common cells of origin for cNF and pNF, but an explanation of specific spatiotemporal differences was lacking.

NF: neurofibroma; GEM: genetically engineered mouse; pNF: plexiform neurofibroma; SC: Schwann cell; NCSC: neural crest stem cell; SCP: Schwann cell precursor; nmSC: non-myelinating Schwann cell; MPNST: malignant peripheral nerve sheath tumor; cNF: cutaneous neurofibroma; SKP: skin-derived neural progenitors; Dhh: desert hedgehog; PLP: myelin proteolipid protein; hiPSCs: human induced pluripotent stem cells.
